# Comparison of the Utility of Total Plasma Fatty Acids Versus those in Cholesteryl Ester, Phospholipid, and Triglyceride as Biomarkers of Fatty Acid Intake

**DOI:** 10.3390/nu11092081

**Published:** 2019-09-03

**Authors:** Jeremy D. Furtado, Jorind Beqari, Hannia Campos

**Affiliations:** Nutritional Biomarker Laboratory, Department of Nutrition, Harvard T.H. Chan School of Public Health, 665 Huntington Ave, Boston, MA 02115, USA

**Keywords:** dietary fat, biomarker, fatty acids, plasma, cholesteryl ester, phospholipid, triglyceride, free fatty acid

## Abstract

Total plasma fatty acids or those in cholesteryl ester and phospholipids are often used to reflect fatty acid intake in epidemiological studies, but their relative performance as biomarkers of intake has not been clearly evaluated within a single population. The assessment of fatty acids in plasma fractions is more labor intensive. Thus, their use as biomarkers of dietary intake needs to be justified. Dietary intake was assessed in 200 population-based controls from a case-control study of diet and heart disease in Costa Rica by a validated food frequency questionnaire (FFQ). Fatty acids in fasting whole plasma and plasma fractions (cholesteryl ester, phospholipid, and triglyceride + free fatty acid) were measured in the 200 controls by the same laboratory using gas chromatography with flame ionization detection (GC-FID). We compared the plasma and plasma fractions data with the FFQ and adipose fatty acid profile using partial Spearman correlations to assess utility as biomarkers of intake and exposure. We found that whole plasma was equally or more strongly correlated with the FFQ and adipose fatty acid profile than either cholesteryl ester or phospholipid in most of the established markers of dietary intake, including dairy (15:0 and 17:0) and seafood (eicosapentaenoic acid and docosahexaenoic acid). Of the three plasma fractions, only fatty acids in the plasma triglyceride + free fatty acid fraction had stronger correlations with dietary intake than whole plasma. In our study population, fatty acids measured in fasting whole plasma perform as good as or better than those measured in plasma fractions as biomarkers for dietary fatty acid intake. Thus, the fractionation of plasma to evaluate long-term fatty acid intake may not be warranted.

## 1. Introduction

Fatty acid biomarkers have been widely used to reflect the long-term intake of fatty acids, especially those not produced within the body, in order to evaluate associations between diet and disease in epidemiological studies [1,2,3,4,5,6,7,8,9,10,11,12,13]. These biomarkers have been measured as a relative fatty acid profile in different types of biospecimens, but the optimal choice is subject to considerable debate. Many studies have reported correlations between blood biomarkers and self-reported dietary intake data from food frequency questionnaires (FFQs) or diet records, and these vary greatly across studies and populations [14,15]. Pools differ in their relative fatty acid profile, which is driven by metabolic control mechanisms and sensitivity to daily intake fluctuations, and thus may differ in their utility to reflect dietary intake. For instance, the main source of dietary fat is triglyceride, excesses of which are stored in adipose. However, the fatty profile of adipose is not entirely analogous to dietary intake due to the presence of endogenously produced fatty acids, preferential oxidation of certain fatty acid classes by tissues during circulation prior to deposition, and biological control mechanisms that effect the uptake and release of fatty acids by adipose tissue. Phospholipids are synthesized on the cytosolic side of the endoplasmic reticulum membrane, and generally have a either a saturated or monounsaturated fatty acid in the sn-1 position and a polyunsaturated fatty acid in the sn-2 position [14]. In addition, cholesteryl esters are manufactured in the body by the transfer of fatty acids from phospholipids to cholesterol either by the enzyme lecithin:cholesterol acyl transferase (LCAT) that transfers a fatty acid from the sn-2 position (usually polyunsaturated) of the phospholipid lecithin (phosphatidylcholine) or by acyl-CoA:cholesterol acyltransferase (ACAT), resulting in a lipid species that is particularly rich in polyunsaturates [14]. 

Adipose tissue is generally considered the fatty acid pool that most closely reflects the usual intake of exogenously sourced fatty acids because it represents very long-term dietary exposure. Indeed, the relative proportion of alpha-linolenic acid in adipose is associated with a reduced risk of myocardial infarction [3]. Long chain omega-3 fatty acids measured in whole blood samples were inversely associated with sudden death [1]. Other studies have focused on individual components within whole blood, such as erythrocytes, whole plasma, and plasma fractions [4,8,9,10,11,12,13]. In a meta-analysis of 19 individual studies looking at the association between coronary heart disease and omega-3 fatty acids from various pools including erythrocytes, whole plasma, plasma fractions (phospholipids, cholesteryl ester, or triglyceride), or adipose [5], the strongest associations with disease were found in omega-3 fatty acids from erythrocytes, total plasma, and plasma phospholipids. This group analyzed omega-6 fatty acids in erythrocyte phospholipids, total plasma, plasma phospholipids, plasma cholesteryl esters, or adipose tissue across a global consortium of 30 prospective observational studies from 13 countries, reporting the strongest associations with reduced risk of disease for increasing linoleic acid as percentage of total fatty acids in total plasma, cholesteryl esters, and adipose tissue [6]. Biomarkers of intake are most useful for fatty acids that are not endogenously synthesized [16]. For example, in whole plasma, medium-chain saturated fatty acids such as tetradecanoic acid (14:0), pentadecanoic acid (15:0) and heptadecanoic acid (17:0), as well as trans-palmitelaidic (16:1n-7t) acid are all markers of dairy consumption; while trans-vaccenic (18:1n-7t) acid in triglycerides and phospholipids, and conjugated linoleic acid (produced by rumen bacteria) in phospholipids significantly correlate with dairy consumption [17,18]. Alpha-linolenic acid and linoleic acid in whole plasma and the plasma cholesteryl ester, triglyceride, and phospholipid fractions all reflect the intake of oils rich in omega-3 and omega-6 fatty acids, respectively, while trans-fatty acids in whole plasma serve as markers of the intake of hydrogenated vegetable oils [19], and longer chain polyunsaturates eicosapentaenoic acid (EPA) and docosahexaenoic acid (DHA) are markers of seafood intake in whole plasma [20,21].

Biospecimens differ in their utility as biomarkers. The measurement of fatty acids in adipose tissue provides a stable and valid estimate of fatty acid profile status for several fatty acids due to its nature as a long-term storage pool [22,23], but access to this tissue requires the acquisition of an adipose aspirate not suitable for most epidemiologic studies. The measurement of fatty acids present in the blood circulation offers a less invasive opportunity to assess the overall fatty acid profile, though fatty acids in this pool are influenced more significantly by recent dietary exposures than is adipose [24]. Blood fatty acids can be determined in whole blood, red blood cells (predominantly from the phospholipid bilayer of the cell membrane) or in plasma [22].

A fair comparison of the ability of each type of biospecimen to reflect long-term intake can be achieved when various types of tissues are simultaneously evaluated in the same population. For example, in the Nurses’ Health Study, a comparison of biomarkers of dietary fat in plasma and erythrocytes found similarly strong correlations with food frequency questionnaire, with favorable spearman correlations for biomarkers of dairy fat 15:0 (*r* = 0.36 for plasma and 0.30 for erythrocytes, respectively) and trans-16:1n-7 (*r* = 0.30 and 0.32) [9], as well as biomarkers of long chain omega-3 fatty acid intake EPA (*r* = 0.31 and 0.32) and DHA (*r* = 0.46 and 0.55) [10]. A study of 200 men and women in Costa Rica showed that diet–tissue correlations for linoleic acid, alpha-linolenic acid, and 18:2-trans fatty acids were strongest with adipose tissue but also strong with plasma, while correlations were poor for red blood cells [22]. In short-term diet studies, compliance with prescribed diets is best assessed by cholesteryl ester or phospholipid fatty acid profile. Based on these results, fatty acids measured in plasma lipid fractions as opposed to whole plasma have been used to reflect long-term dietary fatty acid intake [25]. However, it is unclear whether the use of plasma lipid fractions, a more cumbersome and expensive procedure, is justified. The purpose of this study was to compare the ability of fatty acids in plasma, plasma lipid fractions, and adipose tissue to reflect long-term fatty acid intake within a single population. Using silica resin columns, we isolated three lipid fractions from the plasma of 200 Costa Rican men and women (cholesteryl esters, triglycerides + free (non-esterified) fatty acids, and phospholipids), determined the fatty acid profile of each, and compared the data from these fractions to adipose fatty acid profile and dietary intake assessed by the food frequency questionnaire.

## 2. Materials and Methods

### 2.1. Study Population

The subjects of this study are 101 women and 99 men who were recruited as controls for the Costa Rica Heart Study (CRHS) and who participated in the food frequency questionnaire (FFQ) validation study [26]. CRHS is a population-based, case-control study of nonfatal myocardial infarction (MI) conducted in the central valley area of Costa Rica between 1994 and 2004 [27]. The controls were randomly selected using information from the National Census and Statistics Bureau of Costa Rica and matched to cases of nonfatal acute MI by age (±5 years), sex, and area of residence (county). All subjects provided written informed consent in order to participate in the study. The Ethics Committees of the Harvard School of Public Health and the National Institute of Health Research at the University of Costa Rica approved this study.

### 2.2. Sample Collection

Adipose and fasting blood samples were collected at the subjects’ homes the morning after an overnight fast (minimum 8 h). Subcutaneous adipose tissue biopsy was sampled from the upper buttock with a 16-gauge needle and disposable syringe following procedures described previously [28]. Approximately 2 mg of adipose tissue was stored in Wheaton borosilicate glass vials (Wheaton Science Products, Millville, New Jersey) with solid Teflon caps (DuPont, Wilmington, Delaware) containing 1 mL of hexane:isopropanol (3:2). Fasting blood samples were collected in 10-mL blood collection tubes containing 0.1% ethylenediaminetetraacetic acid (EDTA) and were immediately placed on ice at 4 °C. Within 4 h, blood was centrifuged at 1430× *g* for 20 min at 4 °C to separate plasma from red blood cells and both fractions were stored at −80 °C. Within 6 months, samples were transported on dry ice to the Harvard School of Public Health for long-term storage in the gas phase of liquid nitrogen freezers.

### 2.3. Dietary Assessment

Dietary information was obtained by a semi-quantitative FFQ that inquired about average food consumption during the previous year. This FFQ was based on Willett’s questionnaire and was developed and validated specifically for the Costa Rican population [22,26,29]. Briefly, this FFQ inquired about the intake of 135 food items as well as 20 vitamin, mineral, and food supplements; types of fat used for cooking and frying; and consumption of fried foods in and away from home. Each item had a specified portion size and nine possible coding responses, which ranged from never or less than one per month to six or more per day. The typical Costa Rican diet consisted of rice and beans as the main staple with side dishes containing plantains, chopped vegetables, coleslaw, meat, eggs, or cheese, and tropical fruits. The intake of fish is very low in this population. Fifty-two percent of the population used soybean oil for cooking, 23 percent used palm oil, and the remaining 25 percent used other oils (mainly corn and sunflower) [30].

### 2.4. Fatty Acid Analyses

#### 2.4.1. Lipid Extraction and Fractionation

Lipids were extracted from 150 mcL of plasma by the addition of 6 mL of hexane containing butylated hydroxytoluene (50 mg/L) and 4.5 mL of isopropanol in a 16 mm × 100 mm glass test tube with Teflon-lined caps. The mixture was vortexed for 10 min at an intensity sufficient to ensure complete mixing of all samples and then centrifuged at 1250× *g* for 5 min to sediment the aqueous layer. Then, 4 mL of the organic (upper) layer was removed by glass serological pipette and transferred to a 13 mm × 100 mm disposable borosilicate glass test tube. The solvents were then evaporated to dryness under nitrogen gas flow in a water bath set to 60 °C for approximately 15 min. When evaporation was complete, the lipophilic residue was dissolved in 150 mcL of chloroform.

The gold standard method for the fractionation of lipid classes from human plasma is thin layer chromatography (TLC). However, this technique is more laborious and time-consuming than more recently developed silica column separation techniques. To accommodate the large number of samples we aimed to analyze, we adapted a protocol to isolate three plasma lipid fractions by silica column chromatography that consisted of modifications of several published methods [31,32,33,34,35,36], then validated this protocol against TLC to ensure complete separation. During the evaporation of the plasma extract produced as described above, silica columns (Sep-Pak^®^ Silica 55–105 µm, Glass Syringe 5cc-500 mg; Cat# WAT020585, Waters Corp, Milford, MA, USA) were conditioned by passing the following series of solvents through the columns sequentially in this specific order: 20 mL of hexane, 10 mL of methanol:water (9:1), and 10 mL of chloroform. The flow rate was approximately 5–10 mL/min and 5 mL aliquots of each solvent were loaded onto the column at a time. Columns were not allowed to dry out after conditioning. The 150 mcL sample in chloroform was loaded onto the conditioned column. To collect all of the sample available, the test tube was rinsed with an additional 150 mcL of chloroform, which was also loaded onto the same column. Following the loading of the sample onto the column, a series of solvents was used to elute the various plasma fractions during which a flow rate of 0.2 to 1 mL/min was maintained to ensure proper fractionation. To collect the cholesteryl ester (CE) fraction, a total of 6 mL of light petroleum (Bp. 20–40 °C) was added to the column in 1 mL aliquots. The eluent from these additions was collected in a 13 mm × 100 mm test tube. The second fraction, the combined triglycerides + free (non-esterified) fatty acids (TG + FFA) was eluted using a total of 6 mL of Petroleum Ether (Bp. 30–60 °C): a diethyl-ether:glacial acetic acid mixture (82:18:1) added to the column in 1 mL aliquots and collected in a 13 mm × 100 mm test tube. Lastly, the phospholipid (PL) fraction was collected using a total of 6 mL of a methanol:water mixture (9:1) added to the column in 1 mL aliquots and collected in a 13 mm × 100 mm test tube. Following this final collection, all test tubes were loaded into a water bath set to 60 °C and solvents were evaporated to dryness under nitrogen gas for approximately 30–40 min. The purity of the lipid classes isolated by silica columns was validated by the application of each fraction to thin layer chromatography (TLC) plates. Supplemental Figure S1 shows the successful isolation of plasma fractions by silica columns. Nine spots were loaded on a thin layer chromatography plate: 5 pure standards corresponding to the lipid fractions (cholesteryl ester, triglyceride, free fatty acids, free cholesterol, and phospholipid), an unfractionated plasma sample containing all lipid fractions, and the three fractions produced by the silica column protocol. Free fatty acids are present at very low levels relative to the other lipid species in the whole plasma and the TG + FFA fraction, as expected. They are barely visible to the naked eye but can be identified upon careful inspection. There were no contaminating lipid classes in the column fractions. Supplemental Figure S2 shows the correlation between fatty acid percentages in whole plasma and those from the summed and normalized fractions of fatty acid percentages using silica columns. Because the three lipid fractions studied comprised >90% of all plasma fatty acids [37], the fatty acid profile of the normalized sum of the fractions should be nearly equal to the fatty acid profile for whole plasma if the fatty acids were recovered equally during fractionation. The slope of the regression line was 1.02 with *r*^2^ = 0.99, which shows that recovery was almost complete for each fatty acid.

#### 2.4.2. Lipid Methylation and Peak Identification

Whole plasma and plasma lipid fractions were methylated as described by Zock et al., with modifications [38,39]. Following evaporation, the methanolic sulfuric acid methylation reagent (25 parts methanol: 1 part sulfuric acid) was freshly prepared and 4 mL was added to the test tubes containing the sample residue. The tubes were capped tightly with Teflon-lined caps and heated in an 80 °C water bath for 1 h. Following incubation, samples were removed from heat, uncapped, and 1 mL of distilled, deionized water plus 2 mL of hexane were added. Tubes were recapped and vortexed for 10 min and centrifuged at 1250× *g* for 5 min to sediment the aqueous layer. The hexane (upper) layer was transferred to a new 12 mm × 100 mm test tube by glass Pasteur pipet, washed with 1 mL of distilled, deionized water, and vortexed and centrifuged as described previously. The hexane layer was transferred to a new 12 mm × 100 mm test tube by glass Pasteur pipet. Samples were evaporated to dryness under nitrogen gas flow in a 60 °C water bath for approximately 15 min. Following evaporation, the fatty acid methyl ester residue was dissolved in 100 mcL of iso-octane and transferred to vials for analysis. For adipose tissue, lipids were extracted by dissolution in hexane:isopropanol (3:2), and trans-esterified with methanol and acetyl chloride [40].

Fatty acid methyl esters from adipose, whole plasma, and plasma lipid fractions were analyzed by gas chromatography with flame ionization detection (GC-FID) as previously described [22]. Peak retention times for 45 fatty acids were identified by injecting known standards individually and as a mix, and purity ranges were all above 99 percent (NuCheck Prep, Elysium, MN, USA); Agilent Technologies ChemStation A.08.03 software was used for analysis. The overall coefficients of variation (CVs) for all of the fatty acids in all the lipid fractions studied were monitored by analyzing control pool samples. Supplemental Table S1 shows the mean fatty acid percentages and between-run CVs for the main fatty acids analyzed. Overall, the between-run CVs of the most abundant fatty acids were less than 10% in whole plasma and the plasma lipid fractions, but the CVs for plasma lipid fractions tended to be higher than those of whole plasma. For example, the CV for alpha linolenic acid (ALA) was 4% in whole plasma, 4% in cholesteryl ester, 13% in phospholipid, and 7% in triglyceride + free fatty acid. Acceptable ranges for CVs are dependent on what is being measured, the instrument being used, and the ultimate purpose of the data. Formal acceptable CV ranges have not been established for fatty acid percentages, though, in general, CVs under 20% are good, 20–30% are fair, and over 30% should be used with discretion. Ultimately, the variation inherent in the measurement should be smaller than the variation across the study population in order to be useful. Supplemental Table S1 also includes the study population CVs to allow comparison.

### 2.5. Statistical Analysis

Dietary intake fatty acids are presented as the percentage of total fat intake. Adipose and blood sample fatty acids are presented as a percentage of total fatty acids measured. Descriptive statistics were calculated using Microsoft Excel (2013) and SAS version 9.4 (SAS Institute, Inc., Cary, NC, USA). Partial Spearman correlation coefficients adjusted for age, sex, and body mass index were calculated to determine associations between dietary or adipose fatty acids and those in the different lipid fractions. Correlation coefficients were considered significant at a level of *p* < 0.05. Fisher’s z transformation was used to calculate 95% confidence intervals around each correlation coefficient, and differences between correlation coefficients were considered statistically significant where the 95% confidence interval did not overlap. Comparisons of correlation coefficients were made based on the relative strength of correlation and not statistical significance.

## 3. Results

The average age was 62 and 57 years old in women and men respectively. The mean BMI was 27.2 kg/m^2^ in women and 26 kg/m^2^ and men. Table 1 shows the average main individual fatty acid levels in the diet (FFQ, % of total fat) and mean percentages measured in adipose tissue, total plasma, and plasma lipid fractions (% of total fatty acids). In the diet, three fatty acids make up 78% of the fat consumed in almost equal proportions: palmitic acid (saturated), oleic acid (monounsaturated), and linoleic acid (omega-6 polyunsaturated). Whole plasma and the plasma fraction composed of triglycerides and free fatty acids (TG + FFA) reflect similar patterns with nearly equal proportions of these three fatty acids making up 72 and 77% of the fat, respectively. Adipose tissue also resembles dietary intake data, but with an enrichment in oleic acid (43%) at the expense of linoleic acid (16%). However, the plasma cholesteryl ester (CE) and plasma phospholipid (PL) fractions differ markedly from the others. Over 50% of the fat in CE is linoleic acid, with oleic and palmitic acids making up approximately 20 and 10%, respectively. In plasma PL, palmitic acid makes up approximately 25% of the fat, while linolenic acid makes up 20% and oleic acid only 9%.

**Figure 1:**
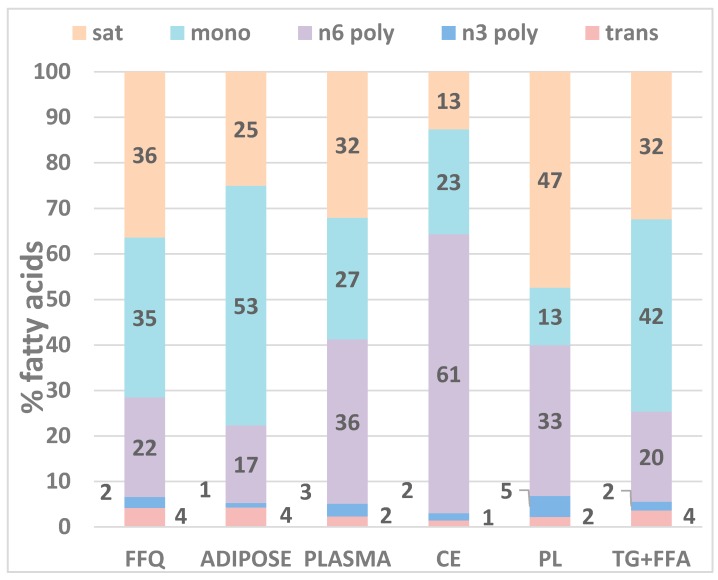
Comparison of the distribution of the main fatty acid groups in the diet (% of total fat intake) and in adipose tissue, total plasma and plasma lipid fractions (% of total fatty acids) separated using silica columns (Sep-Pak^®^ Silica 55–105 µm, Glass Syringe 5cc-500 mg; Cat# WAT020585, Waters Corp, Milford, MA, USA). Sat, saturated fatty acids; mono, monounsaturated fatty acids; n6 poly, omega-6 polyunsaturated fatty acids; n3 poly, omega-3 polyunsaturated fatty acids; trans, trans fatty acids; FFQ, food frequency questionnaire; CE, cholesteryl ester; PL, phospholipid; TG + FFA, triglyceride and free fatty acids combined. Individual fatty acids comprising each group are listed in Table 1.

Figure 1 compares the distributions of the main fatty acid groups in the diet (% of total fat) and in adipose tissue, total plasma, and plasma lipid fractions separated using silica columns (% of total fatty acids). Overall, the TG + FFA fraction most closely resembles the fatty acid profile in FFQ and adipose, followed by whole plasma. Cholesteryl esters are highly enriched in omega-6 polyunsaturates and are depleted in saturated fatty acids, while the phospholipid fraction is enriched in saturated fatty acids and depleted in monounsaturates. These differences reflect unique metabolic processes within the lipid species that result in incorporation of certain fatty acids preferentially over others but are not indicative of their ability to assess dietary intake.

Table 2 shows the partial Spearman correlation coefficients adjusted for age, sex, and body mass index of individual fatty acids in whole plasma and plasma lipid fractions with their intake (FFQ) and adipose tissue fatty acid percentages. The strongest correlations between the plasma fractions and FFQ were found among the exogenous fatty acids, alpha-linolenic acid, linoleic acid and trans fatty acids. The plasma fraction TG + FFA was the most strongly correlated with the FFQ in several of the exogenously-derived fatty acids. For example, the correlation coefficient of this fraction with the FFQ was *r* = 0.49 for linoleic acid and 0.45 for alpha-linolenic acid; compared to *r* = 0.36, 0.35, and 0.17 for linoleic acid and 0.41, 0.31, and 0.15 for alpha-linolenic acid in whole plasma, CE, and PL, respectively. For alpha-linolenic acid, the correlation coefficient between FFQ and phospholipid fatty acids was statistically significantly smaller than for whole plasma (Table 2). Overall, the correlation coefficients between fatty acid percentages measured in CE or PL were no better than and often lower than those obtained for fatty acids measured in whole plasma. Furthermore, the correlations between the FFQ and whole plasma, CE, PL, and TG + FFA were 0.30, 0.26, 0.24, and 0.31 for palmitic acid; 0.19, 0.12, 0.19, and 0.28 for oleic acid; 0.24, 0.26, 0.28, and 0.14 for EPA; and 0.21, 0.21, 0.24, and 0.19 for DHA.

**Figure 2:**
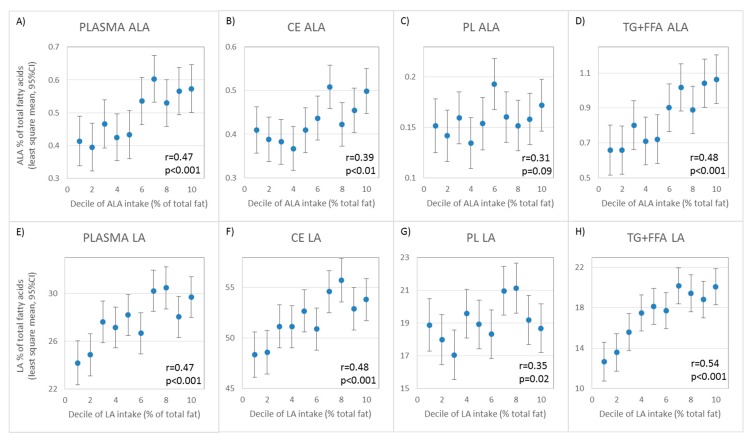
Graphical representation of the correlation between the least-squares mean percentages of alpha-linolenic (18:3n-3) (**A**–**D**) and linoleic acid (18:2n-6) (**E**–**H**) in whole plasma and plasma lipid fractions plotted against their median daily intake (% total fat) in deciles adjusted for age, sex, and body mass index. Bars represent 95% confidence intervals. ALA, alpha linolenic acid; LA, Linoleic Acid.

The correlations between individual fatty acids in whole plasma or the plasma fractions and those in adipose tissue were generally larger and more were statistically significant than the correlations with the FFQ data. Like with FFQ data, TG + FFA compared to whole plasma, CE, and PL was the sample type most strongly correlated with adipose in several important fatty acids, including oleic acid (*r* = 0.48 vs. 0.42, 0.43, and 0.45, respectively), alpha-linolenic (*r* = 0.68 vs. 0.55, 0.34, and 0.26), EPA (*r* = 0.31 vs. 0.28, 0.40, and 0.24), DHA (*r* = 0.47 vs. 0.38, 0.29, and 0.40), and linoleic acid (*r* = 0.70 vs. 0.62, 0.57, and 0.27). TG + FFQ was also the most strongly correlated with adipose for 17:0 (*r* = 0.31) and 18:0 (*r* = 0.22) and was nearly equal to whole plasma in correlation with adipose for monounsaturates, EPA, trans fatty acids, 15:0, and 16:0. As with FFQ, correlation with adipose was generally lower for CE and PL than for TG + FFA and whole plasma, except for EPA, arachidonic acid, and 23:0. The correlations between adipose and phospholipid fatty acids were statistically significantly smaller than for whole plasma for total monounsaturates, alpha-linolenic acid, total omega-6 fatty acids, and linoleic acid (Table 2). The correlations for cholesteryl ester fatty acids were statistically significantly smaller than for whole plasma for 17:0 and gondoic acid. Correlations with adipose for 18:2-trans fatty acids in both phospholipids and cholesteryl esters were statistically significantly lower than for whole plasma. The correlations between the dietary intake of alpha-linolenic (18:3n-3) and linoleic acid (18:2n-6) and their corresponding fatty acid in whole plasma and plasma lipid fractions are illustrated in Figure 2.

## 4. Discussion

This study employs a single population to compare the correlations with dietary intake and adipose tissue fatty acid profiles across several of the more commonly studied plasma sample types: total plasma, plasma cholesteryl ester, plasma triglycerides + free fatty acids, and plasma phospholipids. Previous studies and reviews have compared certain fatty acids across these sample types [9,14,15,17,24,41,42,43,44] but these are either measured in different populations, examine extreme levels of intake outside the realm of typical diets, or involve comparisons across populations that are likely biased by population-specific effects such as differential intake levels, dietary patterns, and even genetic background. The use of a single population allows for a more robust comparison of the relative utility of fatty acids measured in each of the plasma lipid fractions versus whole plasma to reflect dietary intake. However, it must be noted that the results we obtained in our population may not be generalizable to other populations.

Dietary fatty acid intake is typically assessed by food frequency questionnaires (FFQs). While not as detailed as short-term recall and diet records, they are often the method of choice because they can be self-administered relatively quickly and inexpensively, and they reflect average long-term diet, which is more relevant to disease development and progression [16]. However, FFQs rely heavily on accuracy and truthfulness in self-report and validity of the nutrient databases for the foods being assessed. Additionally, they measure intake and not actual exposure within the body because they do not account for variation in absorption, metabolism, and endogenous production. Thus, it is not surprising that our whole plasma and plasma fractions correlate more strongly with adipose tissue than FFQ data. Ultimately, the fatty acid profile in the circulation and lipid stores assessed through tissue analysis may be more relevant than intake in relation to disease. Because FFQ is not a perfect gold standard by which to judge the accuracy of blood biomarkers for assessing true dietary intake, we examined correlations of the blood biomarkers with both FFQ and adipose tissue.

Tissues commonly used to assess fatty acid profile include adipose and blood. Adipose tissue is regarded as the gold-standard because of its very slow turn-over rate [23,45] which reflects long-term exposure and dietary patterns predominantly for the exogenous fatty acids linoleic acid [22,23,45], alpha-linolenic acid [22], and 18:2-trans fatty acids [22]. However, adipose biopsies are invasive and painful to collect. Blood samples can be collected with relative ease and stored for future analysis. For some studies, it may be desirable to assess the fatty acid profile of separate components of blood, commonly fractionating into red blood cells or whole plasma. Within the plasma, fatty acids come mainly from lipoproteins and free (non-esterified) fatty acids circulating in the blood. Free fatty acids are released from adipose tissue via hydrolysis of adipocyte triglycerides by hormone-sensitive lipase (HSL) and are bound to serum albumin for transport to other tissues in the circulation. Free fatty acids make up a very small proportion of total plasma lipids relative to other lipid species. Lipoproteins are comprised of a phospholipid monolayer that surrounds a core of triglycerides and cholesteryl esters. Following a meal, the intestine releases large lipoproteins called chylomicrons that are composed predominantly of triglycerides formed from the fatty acids present in that meal. Therefore, a fasting blood sample is crucial to avoid the influence of recent intake. It has been suggested that, after adipose tissue, phospholipids isolated from plasma provide the most accurate reflection of long-term fatty acid profile and that this lipid pool is superior to total plasma fatty acids because it eliminates fatty acids from triglyceride and cholesteryl esters that are more likely to be sourced from recent dietary ingestion, but this is unclear. Indeed, there is much debate as to which blood fraction provides the most accurate reflection of exposure within the body and dietary intake. The various lipid species in plasma differ in their fatty acid profile, reflecting unique metabolic processes that result in incorporation of certain fatty acids preferentially over others. However, differences in profile do not provide information about the ability of a fatty acid pool to reflect diet. A fatty acid could be less abundant in one lipid species but more correlated with diet than in another pool in which it is more abundant. Red blood cells were thought to provide a better long-term picture of fatty acid profile than plasma due to the cells ~3 months residence time in circulation with several fatty acids reported to have half-lives in the pool of approximately 30 days [24]. However, cell membranes are dynamic structures that exchange phospholipids throughout their lifetime and the fatty acids that comprise the membranes are under physiological regulation such that the profile of fatty acids in the membrane are not entirely reflective of dietary intake [46]. Further, lipid peroxidation of fatty acids may occur during sample collection and processing, even during ultra-low temperature storage, due to the presence of reactive iron species and some heme moieties, thus the measured sample may not be reflective of the true fatty acid profile within the body. It has been argued that whole plasma or serum are less useful as they only reflect recent intake over the past several days. However, in the fasting state the lipids in plasma or serum are sourced from a combination of exogenous and endogenous sources. Composed predominantly of a mixture of phospholipids, triglycerides, and cholesteryl esters from lipoproteins and free fatty acids released from adipose tissue stores, total plasma or serum offers the advantage of sourcing fatty acids from multiple fatty acid depots which may overcome limitations of the specific lipid species imparted by their turnover rates and levels of physiological control.

Indeed, we have found that fasting total plasma fatty acid profile is better correlated with both FFQ and adipose fatty acid profile than are the fatty acid profiles for cholesteryl ester or phospholipid. The data in our study cannot definitively address the reasons why whole plasma shows equal or stronger correlations in most cases compared to the phospholipid and cholesteryl ester fractions, but we hypothesize that this is likely due to the averaging of the strengths and weakness of the individual fractions. For example, the correlation with ALA in adipose is 0.68 for TG + FFA but only 0.26 for PL, and when you combine these together with CE as a whole plasma the correlation is 0.55. Unexpectedly, we found that the TG + FFA fraction was equal to or superior over the whole plasma fraction in many important biomarkers of intake and exposure. Others have reported good correlations between adipose tissue fatty acid profile and free fatty acid profile [47], which may stem from plasma free fatty acids present in the fasting state arising by lipolysis of adipose tissue. But it has been suggested that fatty acids are selectively mobilized from the adipose tissue into plasma [48] and therefore do not reflect dietary intake or adipose fatty acid profile. Indeed, some studies do show very poor correlations [49], and it has been shown that FID detection is not ideal for free fatty acids, especially at low levels. Our study examined combined triglycerides + free fatty acids, thus the valuable lipid species could be the triglyceride. In a recent study, plasma triglyceride was the strongest marker of dairy intake [17]. Literature examining the association between the fatty acid profiles of plasma triglyceride and adipose is relatively sparse and discrepant [50,51].

The strengths of this study include the measurement of all lipid depots in the same participants, the use of healthy participants consuming a free-living diet typical of many Western countries, a sample size of 200 individuals, and the lab assays being conducted all in the same laboratory using the same instrumentation. Our study is limited in that the results may not be applicable to samples stored under conditions or lengths of time that differ from ours (−80 °C for up to 6 months then in liquid nitrogen for 20 years) because changes in fatty acid profile can occur during storage that vary with temperature and time, as well as across blood fraction. Our study used samples stored at conditions that are typical of many large cohorts, so the results are useful to many study designs. Additionally, our results may not extend to other populations with different dietary patterns or genetic background. It should be repeated in other populations representing different dietary patterns to assess generalizability, especially in the case of studies comparing different populations. It is also possible that the correlations we found may only apply to healthy populations and not to individuals in diseased states.

## 5. Conclusions

Our data indicate that, for our study population, fasting whole plasma is equivalent or superior to cholesteryl ester and phospholipid for important fatty acid biomarkers of intake and exposure such as oleic acid, alpha-linolenic acid, EPA, DHA, linoleic acid, trans fatty acids, and medium-chain saturates 14:0, 15:0, and 17:0, which are markers of dairy intake. Therefore, the additional time, expense, and increase in measurement error imparted by the isolation of cholesteryl ester and phospholipid fractions is not warranted. Moreover, the plasma fraction with the greatest utility as a biomarker of intake and exposure is actually triglycerides + free fatty acids. However, the marginal increase in strength of correlations in this fraction over whole plasma is not worth the cost. Therefore, assessing fasting whole plasma or serum as a biomarker of fatty acid intake or exposure status is recommended over plasma fractions.

## Figures and Tables

**Table 1:** Mean (95% confidence interval) dietary fatty acids (as % of total fat) obtained by the food frequency questionnaire and individual fatty acid percentages in adipose tissue, total plasma, and plasma lipid fractions among 200 participants in the Costa Rica Heart Study.

Carbon Name	Common Name	Total			Plasma Fractions		
		FFQ	Adipose	Plasma	CE	PL	TG + FFA
**saturated**		**36.5 (35.5, 37.5)**	**25.0 (24.5, 25.5)**	**32.1 (31.7, 32.5)**	**12.6 (12.3, 12.9)**	**47.4 (46.8, 47.9)**	**32.4 (31.8, 32.9)**
13:0		0.02 (0.01, 0.02)	0.01 (0.00, 0.01)	0.01 (0.01, 0.01)	0.01 (0.01, 0.01)	0.01 (0.01, 0.01)	0.00
14:0	Myristic Acid	2.88 (2.70, 3.05)	1.14 (1.07, 1.20)	0.60 (0.56, 0.63)	0.23 (0.21, 0.25)	0.14 (0.13, 0.15)	0.87 (0.82, 0.93)
15:0		0.31 (0.30, 0.33)	0.22 (0.21, 0.23)	0.16 (0.15, 0.17)	0.10 (0.09, 0.10)	0.12 (0.11, 0.13)	0.19 (0.18, 0.20)
16:0	Palmitic Acid	23.7 (22.9, 24.4)	20.6 (20.2, 21.0)	22.0 (21.7, 22.4)	10.2 (10.0, 10.4)	25.0 (24.4, 25.5)	24.4 (23.9, 24.8)
17:0	Margaric Acid	0.33 (0.32, 0.35)	0.20 (0.20, 0.21)	0.31 (0.30, 0.32)	0.16 (0.15, 0.18)	0.44 (0.42, 0.45)	0.40 (0.39, 0.42)
18:0	Stearic Acid	7.84 (7.64, 8.04)	2.65 (2.53, 2.78)	7.56 (7.43, 7.68)	1.72 (1.57, 1.87)	17.4 (17.0, 17.7)	6.20 (5.98, 6.42)
19:0		0.08 (0.08, 0.09)	0.09 (0.09, 0.10)	0.09 (0.08, 0.09)	0.09 (0.07, 0.10)	0.14 (0.13, 0.16)	0.11 (0.11, 0.12)
20:0	Arachidic Acid	0.21 (0.21, 0.22)	0.09 (0.08, 0.10)	0.20 (0.19, 0.20)	0.08 (0.07, 0.10)	0.54 (0.52, 0.56)	0.12 (0.11, 0.13)
22:0	Behenic Acid	0.10 (0.09, 0.11)	0.00	0.47 (0.45, 0.49)	0.03 (0.02, 0.03)	1.50 (1.44, 1.57)	0.04 (0.04, 0.05)
23:0		0.00	0.00	0.23 (0.22, 0.24)	0.00 (0.00, 0.01)	0.77 (0.73, 0.81)	0.02 (0.02, 0.02)
24:0	Lignoceric Acid	0.00	0.02 (0.02, 0.03)	0.42 (0.40, 0.44)	0.01 (0.00, 0.01)	1.43 (1.34, 1.52)	0.06 (0.05, 0.07)
**monounsaturated**		**34.9 (33.8, 36.0)**	**52.6 (52.0, 53.1)**	**26.7 (26.2, 27.2)**	**23.0 (22.4, 23.6)**	**12.6 (12.3, 13.0)**	**42.2 (41.7, 42.8)**
14:1n-5c	Myristoleic Acid	0.21 (0.20, 0.22)	0.18 (0.17, 0.20)	0.03 (0.03, 0.04)	0.03 (0.03, 0.04)	0.03 (0.02, 0.03)	0.06 (0.05, 0.06)
15:1n-5c		0.00	0.00	0.01 (0.01, 0.01)	0.04 (0.03, 0.06)	0.06 (0.04, 0.08)	0.02 (0.02, 0.03)
16:1n-7c	Palmitoleic Acid	1.41 (1.36, 1.47)	6.75 (6.45, 7.04)	2.42 (2.29, 2.55)	2.67 (2.49, 2.85)	0.47 (0.43, 0.50)	3.34 (3.19, 3.50)
18:1n-9c	Oleic Acid	31.7 (30.7, 32.8)	42.9 (42.5, 43.3)	21.6 (21.2, 22.0)	18.9 (18.4, 19.4)	8.70 (8.41, 8.99)	35.7 (35.2, 36.2)
18:1n-7c		1.36 (1.32, 1.41)	2.29 (2.22, 2.35)	1.82 (1.77, 1.86)	1.29 (1.26, 1.33)	1.37 (1.33, 1.41)	2.66 (2.59, 2.73)
20:1n-9c	Gondoic Acid	0.17 (0.16, 0.18)	0.43 (0.42, 0.45)	0.17 (0.17, 0.18)	0.02 (0.02, 0.02)	0.18 (0.17, 0.18)	0.33 (0.32, 0.34)
24:1n-9c	Nervonic Acid	0.01 (0.00, 0.01)	0.00	0.61 (0.58, 0.64)	0.06 (0.06, 0.07)	1.82 (1.75, 1.90)	0.13 (0.09, 0.17)
**omega-3 polyunsaturated**		**2.41 (2.29, 2.52)**	**0.98 (0.95, 1.01)**	**2.76 (2.65, 2.87)**	**1.58 (1.50, 1.66)**	**4.64 (4.44, 4.84)**	**1.89 (1.79, 1.98)**
18:3n-3c	Alpha-linolenic Acid (ALA)	1.95 (1.85, 2.05)	0.63 (0.61, 0.66)	0.49 (0.47, 0.52)	0.43 (0.41, 0.44)	0.16 (0.15, 0.17)	0.84 (0.80, 0.89)
20:5n-3c	EPA	0.15 (0.13, 0.18)	0.02 (0.02, 0.03)	0.38 (0.35, 0.41)	0.53 (0.48, 0.58)	0.50 (0.46, 0.54)	0.17 (0.15, 0.19)
22:5n-3c	DPA	0.08 (0.07, 0.08)	0.19 (0.18, 0.20)	0.42 (0.40, 0.44)	0.04 (0.03, 0.04)	0.83 (0.79, 0.87)	0.32 (0.29, 0.34)
22:6n-3c	DHA	0.23 (0.20, 0.26)	0.14 (0.13, 0.15)	1.47 (1.40, 1.54)	0.58 (0.55, 0.61)	3.16 (3.01, 3.31)	0.56 (0.52, 0.60)
**omega-6 polyunsaturated**		**21.9 (20.9, 23.0)**	**17.1 (16.6, 17.6)**	**36.1 (35.4, 36.8)**	**61.3 (60.6, 62.1)**	**33.1 (32.6, 33.7)**	**19.8 (19.1, 20.5)**
18:2n-6c	Linoleic Acid (LA)	21.5 (20.5, 22.5)	15.8 (15.3, 16.2)	27.7 (27.1, 28.3)	52.0 (51.3, 52.7)	19.1 (18.6, 19.6)	17.4 (16.7, 18.0)
18:3n-6c	Gamma-linolenic Acid (GLA)	0.04 (0.03, 0.04)	0.05 (0.05, 0.05)	0.38 (0.36, 0.40)	0.87 (0.82, 0.92)	0.11 (0.09, 0.12)	0.30 (0.28, 0.32)
20:2n-6c		0.05 (0.04, 0.05)	0.24 (0.23, 0.25)	0.27 (0.26, 0.28)	0.04 (0.04, 0.05)	0.40 (0.39, 0.42)	0.30 (0.29, 0.31)
20:3n-6c	Dihomogammalinolenic Acid (DHGLA)	0.04 (0.04, 0.04)	0.33 (0.32, 0.35)	1.75 (1.70, 1.81)	1.03 (0.99, 1.07)	3.74 (3.61, 3.87)	0.40 (0.38, 0.42)
20:4n-6c	Arachidonic Acid (AA)	0.26 (0.25, 0.28)	0.48 (0.46, 0.50)	5.76 (5.52, 6.01)	7.31 (6.96, 7.65)	9.26 (8.92, 9.61)	1.22 (1.16, 1.28)
22:2n-6c		0.00	0.01 (0.01, 0.01)	0.02 (0.02, 0.02)	0.02 (0.02, 0.02)	0.05 (0.04, 0.05)	0.02 (0.01, 0.02)
22:4n-6c	Adrenic Acid	0.04 (0.03, 0.04)	0.21 (0.20, 0.22)	0.25 (0.24, 0.26)	0.03 (0.03, 0.03)	0.48 (0.47, 0.50)	0.22 (0.21, 0.23)
**trans fatty acids**		**4.19 (3.94, 4.43)**	**4.33 (4.21, 4.45)**	**2.35 (2.28, 2.42)**	**1.45 (1.41, 1.50)**	**2.21 (2.14, 2.28)**	**3.66 (3.55, 3.78)**
14:1n-5t	Myristelaidic Acid	0.00	0.00	0.01 (0.01, 0.01)	0.01 (0.01, 0.01)	0.05 (0.04, 0.06)	0.00
16:1n-7t	Palmitelaidic Acid	0.19 (0.18, 0.21)	0.17 (0.16, 0.17)	0.12 (0.12, 0.13)	0.07 (0.06, 0.07)	0.15 (0.14, 0.16)	0.11 (0.11, 0.12)
18:1-trans		2.38 (2.26, 2.51)	1.96 (1.87, 2.05)	1.02 (0.96, 1.07)	0.33 (0.32, 0.35)	1.02 (0.98, 1.07)	1.84 (1.75, 1.94)
18:2-trans		1.24 (1.12, 1.35)	1.10 (1.06, 1.14)	0.55 (0.54, 0.57)	0.56 (0.53, 0.58)	0.50 (0.47, 0.52)	0.80 (0.77, 0.82)
18:2n-7c	c9, t11 conjugated linoleic acid (CLA)	0.33 (0.31, 0.36)	0.54 (0.42, 0.66)	0.25 (0.24, 0.26)	0.20 (0.19, 0.22)	0.16 (0.15, 0.17)	0.39 (0.37, 0.40)
20:1n-9t		0.04 (0.03, 0.05)	0.02 (0.01, 0.02)	0.00	0.00	0.01 (0.01, 0.02)	0.00

FFQ, food frequency questionnaire; CE, cholesteryl ester; PL, phospholipid; TG + FFA, triglyceride plus free fatty acids. EPA, eicosapentaenoic acid; DPA, docosapentaenoic acid; DHA, docosahexaenoic acid. Plasma lipid fractions separated using silica columns (Sep-Pak^®^ Silica 55–105 µm, Glass Syringe 5cc-500 mg; Waters Corp Cat# WAT020585).

**Table 2:** Partial Spearman correlation coefficients (95% confidence intervals) adjusted for age, sex, and body mass index of individual fatty acids in whole plasma and plasma lipid fractions with their intake (FFQ, Panel A) and adipose tissue fatty acid percentages (Panel B). *p*-value < 0.05 for all *r* > 0.14.

Panel A					
Carbon Name	Common Name	Correlation with FFQ			
		Plasma	CE	PL	TG + FFA
**saturated**		**0.23 (0.09, 0.36)**	**0.19 (0.04, 0.32)**	**0.12 (−0.02, 0.26)**	**0.27 (0.13, 0.40)**
13:0		−0.12 (−0.26, 0.02)	−0.02 (−0.17, 0.12)	0.03 (−0.12, 0.17)	−0.05 (−0.19, 0.10)
14:0	Myristic Acid	0.07 (−0.07, 0.21)	−0.01 (−0.15, 0.13)	0.03 (−0.11, 0.17)	0.02 (−0.13, 0.16)
15:0		0.23 (0.09, 0.36)	0.16 (0.01, 0.29)	0.15 (0.01, 0.29)	0.20 (0.06, 0.33)
16:0	Palmitic Acid	0.30 (0.16, 0.42)	0.26 (0.12, 0.39)	0.24 (0.10, 0.37)	0.31 (0.18, 0.44)
17:0	Margaric Acid	0.06 (−0.08, 0.20)	0.09 (−0.05, 0.23)	0.02 (−0.12, 0.16)	0.10 (−0.04, 0.24)
18:0	Stearic Acid	0.05 (−0.09, 0.19)	0.03 (−0.11, 0.17)	−0.01 (−0.15, 0.14)	0.05 (−0.10, 0.19)
19:0		0.10 (−0.05, 0.24)	0.02 (−0.13, 0.16)	0.03 (−0.11, 0.17)	0.05 (−0.10, 0.19)
20:0	Arachidic Acid	0.08 (−0.06, 0.22)	0.04 (−0.10, 0.18)	0.02 (−0.12, 0.16)	0.03 (−0.11, 0.17)
22:0	Behenic Acid	0.04 (−0.10, 0.18)	0.05 (−0.09, 0.19)	0.12 (−0.02, 0.26)	0.09 (−0.05, 0.23)
23:0		0.11 (−0.04, 0.24)	−0.14 (−0.28, 0.00)	0.09 (−0.05, 0.23)	0.07 (−0.07, 0.21)
24:0	Lignoceric Acid	.	.	.	.
**monounsaturated**		**0.14 (0.00, 0.28)**	**0.10 (−0.05, 0.24)**	**0.17 (0.03, 0.30)**	**0.23 (0.09, 0.36)**
14:1n-5c	Myristoleic Acid	0.13 (−0.02, 0.26)	−0.06 (−0.20, 0.08)	−0.01 (−0.15, 0.13)	0.07 (−0.07, 0.21)
15:1n-5c		.	.	.	.
16:1n-7c	Palmitoleic Acid	0.05 (−0.09, 0.19)	0.05 (−0.1, 0.19)	0.03 (−0.12, 0.17)	0.02 (−0.13, 0.16)
18:1n-9c	Oleic Acid	0.19 (0.05, 0.32)	0.12 (−0.02, 0.26)	0.19 (0.05, 0.32)	0.28 (0.15, 0.41)
18:1n-7c		−0.06 (−0.20, 0.09)	−0.09 (−0.23, 0.05)	−0.01 (−0.15, 0.14)	−0.06 (−0.20, 0.08)
20:1n-9c	Gondoic Acid	0.10 (−0.05, 0.23)	−0.09 (−0.23, 0.06)	0.00 (−0.14, 0.14)	0.08 (−0.06, 0.22)
24:1n-9c	Nervonic Acid	−0.00 (−0.14, 0.14)	−0.00 (−0.14, 0.14)	0.00 (−0.14, 0.14)	−0.08 (−0.22, 0.06)
**omega-3 polyunsaturated**		**0.27 (0.13, 0.39)**	**0.28 (0.15, 0.41)**	**0.22 (0.09, 0.35)**	**0.34 (0.21, 0.46)**
18:3n-3c	Alpha-linolenic Acid (ALA)	0.41 (0.29, 0.52)	0.31 (0.18, 0.44)	0.15 (0.01, 0.28) †	0.45 (0.33, 0.56)
20:5n-3c	EPA	0.24 (0.11, 0.37)	0.26 (0.13, 0.39)	0.28 (0.15, 0.41)	0.14 (−0.00, 0.27)
22:5n-3c	DPA	0.10 (−0.04, 0.24)	−0.02 (−0.16, 0.13)	0.13 (−0.01, 0.27)	0.12 (−0.03, 0.25)
22:6n-3c	DHA	0.21 (0.08, 0.35)	0.21 (0.07, 0.34)	0.24 (0.10, 0.37)	0.19 (0.05, 0.33)
**omega-6 polyunsaturated**		**0.36 (0.23, 0.47)**	**0.40 (0.35, 0.57)**	**0.24 (0.10, 0.37)**	**0.47 (0.35, 0.57)**
18:2n-6c	Linoleic Acid (LA)	0.36 (0.23, 0.48)	0.35 (0.22, 0.47)	0.17 (0.03, 0.30)	0.49 (0.37, 0.59)
18:3n-6c	Gamma-linolenic Acid (GLA)	0.07 (−0.07, 0.21)	0.03 (−0.11, 0.17)	0.05 (−0.09, 0.19)	0.09 (−0.05, 0.23)
20:2n-6c		−0.11 (−0.25, 0.03)	−0.01 (−0.16, 0.13)	−0.07 (−0.21, 0.07)	−0.05 (−0.19, 0.09)
20:3n-6c	Dihomogammalinolenic Acid (DHGLA)	−0.14 (−0.27, 0.01)	−0.13 (−0.27, 0.01)	−0.12 (−0.26, 0.02)	−0.12 (−0.26, 0.02)
20:4n-6c	Arachidonic Acid (AA)	0.14 (−0.01, 0.27)	0.15 (0.01, 0.29)	0.20 (0.06, 0.33)	0.13 (−0.02, 0.26)
22:2n-6c		.	.	.	.
22:4n-6c	Adrenic Acid	0.02 (−0.12, 0.16)	0.02 (−0.12, 0.16)	0.02 (−0.12, 0.16)	0.03 (−0.11, 0.17)
**trans fatty acids**		**0.28 (0.14, 0.40)**	**0.36 (0.22, 0.47)**	**0.22 (0.08, 0.35)**	**0.21 (0.07, 0.34)**
14:1n-5t	Myristelaidic Acid	.	.	.	.
16:1n-7t	Palmitelaidic Acid	0.15 (0.01, 0.29)	0.14 (0.00, 0.28)	0.17 (0.03, 0.31)	0.20 (0.06, 0.33)
18:1-trans		0.20 (0.06, 0.34)	0.23 (0.09, 0.36)	0.18 (0.04, 0.31)	0.16 (0.01, 0.29)
18:2-trans		0.45 (0.32, 0.55)	0.34 (0.21, 0.46)	0.31 (0.17, 0.43)	0.43 (0.30, 0.53)
18:2n-7c	c9, t11 conjugated linoleic acid (CLA)	−0.01 (−0.15, 0.14)	−0.01 (−0.16, 0.13)	0.04 (−0.11, 0.18)	0.08 (−0.06, 0.22)
20:1n-9t		0.29 (0.16, 0.42)	0.25 (0.11, 0.38)	0.20 (0.11, 0.38)	0.21 (0.07, 0.34)
**Panel B**					
**Carbon Name**	**Common Name**	**Correlation with Adipose**			
		**Plasma**	**CE**	**PL**	**TG + FFA**
**saturated**		**0.34 (0.21, 0.46)**	**0.12 (−0.02, 0.26)**	**0.17 (0.02, 0.30)**	**0.38 (0.25, 0.50)**
13:0		−0.14 (−0.27, 0.00)	0.11 (−0.03, 0.25)	−0.03 (−0.17, 0.11)	−0.02 (−0.16, 0.13)
14:0	Myristic Acid	0.17 (0.03, 0.30)	0.24 (0.10, 0.37)	0.06 (−0.08, 0.20)	0.09 (−0.05, 0.23)
15:0		0.41 (0.28, 0.52)	0.35 (0.22, 0.47)	0.22 (0.08, 0.35)	0.39 (0.26, 0.50)
16:0	Palmitic Acid	0.40 (0.27, 0.51)	0.18 (0.04, 0.31)	0.26 (0.13, 0.39)	0.46 (0.34, 0.57)
17:0	Margaric Acid	0.40 (0.28, 0.51)	0.13 (−0.02, 0.26) †	0.28 (0.14, 0.41)	0.32 (0.18, 0.44)
18:0	Stearic Acid	0.09 (−0.05, 0.23)	0.01 (−0.13, 0.15)	−0.07 (−0.21, 0.08)	0.22 (0.08, 0.35)
19:0		0.30 (0.16, 0.42)	0.16 (0.02, 0.30)	0.07 (−0.08, 0.21)	0.24 (0.10, 0.37)
20:0	Arachidic Acid	0.02 (−0.13, 0.16)	0.16 (0.01, 0.29)	−0.01 (−0.16, 0.13)	0.29 (0.15, 0.41)
22:0	Behenic Acid	−0.05 (−0.19, 0.10)	−0.06 (−0.20, 0.09)	0.06 (−0.09, 0.20)	−0.06 (−0.20, 0.08)
23:0		−0.08 (−0.22, 0.06)	0.11 (−0.03, 0.25)	0.16 (0.02, 0.29)	0.10 (−0.05, 0.23)
24:0	Lignoceric Acid	.	.	.	.
**monounsaturated**		**0.65 (0.56, 0.72)**	**0.51 (0.39, 0.61)**	**0.37 (0.24, 0.49) †**	**0.52 (0.41, 0.62)**
14:1n-5c	Myristoleic Acid	0.14 (−0.01, 0.27)	0.11 (−0.03, 0.25)	0.18 (0.04, 0.31)	0.12 (−0.02, 0.26)
15:1n-5c		0.10 (−0.05, 0.23)	0.02 (−0.12, 0.16)	0.04 (−0.10, 0.18)	0.09 (−0.05, 0.23)
16:1n-7c	Palmitoleic Acid	0.38 (0.25, 0.49)	0.34 (0.20, 0.46)	0.29 (0.15, 0.41)	0.43 (0.31, 0.54)
18:1n-9c	Oleic Acid	0.42 (0.30, 0.53)	0.43 (0.31, 0.54)	0.45 (0.33, 0.55)	0.48 (0.36, 0.58)
18:1n-7c		0.40 (0.28, 0.51)	0.30 (0.17, 0.43)	0.25 (0.11, 0.38)	0.41 (0.28, 0.52)
20:1n-9c	Gondoic Acid	0.23 (0.09, 0.36)	−0.10 (−0.24, 0.04) †	0.10 (−0.04, 0.24)	0.22 (0.07, 0.35)
24:1n-9c	Nervonic Acid	0.09 (−0.05, 0.23)	0.02 (−0.12, 0.16)	0.02 (−0.13, 0.16)	0.04 (−0.10, 0.18)
**omega-3 polyunsaturated**		**0.41 (0.28, 0.52)**	**0.33 (0.19, 0.45)**	**0.29 (0.15, 0.42)**	**0.54 (0.42, 0.63)**
18:3n-3c	Alpha-linolenic Acid (ALA)	0.55 (0.44, 0.64)	0.34 (0.21, 0.46)	0.26 (0.12, 0.39) †	0.68 (0.60, 0.75)
20:5n-3c	EPA	0.28 (0.15, 0.41)	0.40 (0.27, 0.51)	0.24 (0.10, 0.37)	0.31 (0.18, 0.44)
22:5n-3c	DPA	0.29 (0.15, 0.41)	0.11 (−0.03, 0.25)	0.27 (0.13, 0.40)	0.34 (0.21, 0.46)
22:6n-3c	DHA	0.38 (0.25, 0.49)	0.29 (0.16, 0.42)	0.40 (0.27, 0.51)	0.47 (0.35, 0.57)
**omega-6 polyunsaturated**		**0.57 (0.46, 0.65)**	**0.56 (0.46, 0.65)**	**0.27 (0.13, 0.40) †**	**0.67 (0.58, 0.74)**
18:2n-6c	Linoleic Acid (LA)	0.62 (0.53, 0.70)	0.57 (0.46, 0.66)	0.27 (0.14, 0.40) †	0.70 (0.61, 0.76)
18:3n-6c	Gamma-linolenic Acid (GLA)	0.23 (0.09, 0.36)	0.20 (0.06, 0.33)	0.18 (0.04, 0.31)	0.28 (0.14, 0.40)
20:2n-6c		0.35 (0.22, 0.47)	−0.02 (−0.17, 0.12) †	0.28 (0.15, 0.41)	0.30 (0.16, 0.42)
20:3n-6c	Dihomogammalinolenic Acid (DHGLA)	0.26 (0.12, 0.38)	0.29 (0.15, 0.41)	0.30 (0.17, 0.43)	0.19 (0.05, 0.32)
20:4n-6c	Arachidonic Acid (AA)	0.37 (0.24, 0.49)	0.37 (0.24, 0.49)	0.42 (0.30, 0.53)	0.31 (0.18, 0.43)
22:2n-6c		−0.08 (−0.22, 0.06)	0.06 (−0.08, 0.20)	0.01 (−0.13, 0.15)	−0.02 (−0.16, 0.13)
22:4n-6c	Adrenic Acid	0.22 (0.08, 0.35)	−0.03 (−0.17, 0.11)	0.17 (0.03, 0.30)	0.25 (0.11, 0.37)
**trans fatty acids**		**0.54 (0.43, 0.63)**	**0.34 (0.21, 0.46)**	**0.40 (0.28, 0.52)**	**0.54 (0.43, 0.63)**
14:1n-5t	Myristelaidic Acid	0.01 (−0.14, 0.15)	0.12 (−0.03, 0.25)	−0.09 (−0.23, 0.06)	0.03 (−0.11, 0.18)
16:1n-7t	Palmitelaidic Acid	0.39 (0.26, 0.50)	0.17 (0.03, 0.31)	0.29 (0.15, 0.41)	0.25 (0.11, 0.38)
18:1-trans		0.45 (0.33, 0.56)	0.37 (0.24, 0.49)	0.38 (0.25, 0.49)	0.47 (0.35, 0.57)
18:2-trans		0.52 (0.40, 0.61)	0.27 (0.13, 0.39) †	0.21 (0.07, 0.34) †	0.61 (0.51, 0.69)
18:2n-7c	c9, t11 conjugated linoleic acid (CLA)	0.01 (−0.14, 0.15)	0.02 (−0.12, 0.16)	−0.06 (−0.20, 0.08)	−0.06 (−0.20, 0.08)
20:1n-9t		0.36 (0.23, 0.47)	0.27 (0.13, 0.40)	0.19 (0.05, 0.32)	0.43 (0.31, 0.54)

In Panel A, † *p* < 0.05 for difference compared to the correlation of whole plasma with FFQ. In In Panel B, † *p* < 0.05 for difference compared to the correlation of whole plasma with adipose. CE, cholesteryl ester; PL, phospholipid; TG + FFA, triglyceride plus free fatty acids. Plasma lipid fractions separated using silica columns (Sep-Pak^®^ Silica 55–105 µm, Glass Syringe 5cc-500 mg; Waters Corp Cat# WAT020585).

## Supplementary Materials

The following are available online at https://www.mdpi.com/2072-6643/11/9/2081/s1, Figure S1: Thin layer chromatography plate (Analtech Uniplate Cat# 02011) confirming successful resolution of plasma fractions by silica columns. From left to right: migration of pure standards of cholesteryl ester (CE; cholesteryl heptadecanoate, NuChek Prep Cat# CH-816), triglyceride (TG; tripentadecanoin, NuChek Prep Cat# T-145), free fatty acid (FFA; pentadecanoic acid, NuChek PrepCat# N-15-A), free cholesterol (FC; NuChek Prep Cat# CH-800), and phospholipid (PL; Phosphotidyl choline; Avanti Polar Lipids Cat# 850360C); whole plasma (WP); and the three plasma fractions prepared by silica columns (Sep-Pak® Silica 55–105 µm, Glass Syringe 5cc-500 mg; Waters Corp Cat# WAT020585). Figure S2: Correlation between the fatty acid levels measured in whole plasma and those obtained from the sum of all fatty acids from the silica column fractions. The smaller graph shows only those fatty acids whose area comprises <2% of total plasma fatty acids. Table S1: Mean levels and between run coefficients of variation (CV) % for the major fatty acids assessed in the quality control pools of whole plasma and plasma lipid fractions.
