# Supplementary material for: Comparison of the Utility of Total Plasma Fatty Acids Versus those in Cholesteryl Ester, Phospholipid, and Triglyceride as Biomarkers of Fatty Acid Intake
Source: Nutrients. 2019 Sep 3;11(9):2081. doi: 10.3390/nu11092081 (PMC6770493; doi:10.3390/nu11092081)
Supplement: Supplementary file 1 [file nutrients-11-02081-s001.pdf]

Supplemental:

Table 1. Mean levels and between run coefficients of variation (CV) % for the major fatty acids assessed in the quality control pools of whole plasma and plasma lipid fractions.

		**PLASMA**			**CE**			**PL**			**TG+FFA**		
		**CONTROL**		**POP**	**CONTROL**		**POP**	**CONTROL**		**POP**	**CONTROL**		**POP**
Carbon Name	Common Name	**MEAN**	**CV %**	**CV%**	**MEAN**	**CV %**	**CV%**	**MEAN**	**CV %**	**CV%**	**MEAN**	**CV %**	**CV%**
saturated													
13:0		0	NA	201%	0	NA	780%	0	NA	707%	0	NA	332%
14:0	Myristic Acid	0.69	25%	43%	0.49	45%	58%	0.26	42%	60%	1.25	25%	46%
15:0		0.17	17%	32%	0.13	23%	38%	0.15	13%	38%	0.21	17%	32%
16:0	Palmitic Acid	20.88	6%	11%	10.16	9%	14%	26.07	6%	16%	22.97	6%	14%
17:0	Margaric Acid	0.29	5%	22%	0.1	46%	64%	0.4	14%	26%	0.37	19%	26%
18:0	Stearic Acid	7.84	7%	12%	1.15	24%	62%	14.36	10%	16%	6.43	23%	25%
19:0		0.08	20%	33%	0.04	139%	125%	0.11	33%	64%	0.12	63%	52%
20:0	Arachidic Acid	0.26	13%	23%	0.21	50%	142%	0.51	16%	27%	0.3	28%	66%
22:0	Behenic Acid	0.6	19%	31%	0.03	129%	96%	1.2	21%	32%	0.06	59%	61%
23:0		0.26	17%	33%	0	NA	212%	0.52	23%	39%	0	NA	131%
24:0	Lignoceric Acid	0.5	17%	38%	0	NA	225%	0.94	30%	45%	0.06	60%	143%
	monounsaturated												
14:1n-5c	Mysristoleic Acid	0.04	43%	61%	0	NA	112%	0	NA	202%	0.03	58%	60%
15:1n-5c		0.01	60%	65%	0.08	160%	185%	0.04	301%	219%	0.06	168%	191%
16:1n-7c	Palmitoleic Acid	1.67	8%	39%	2.28	15%	49%	0.44	10%	54%	2.82	20%	33%
18:1n-9c	Oleic Acid	17.76	2%	13%	17.22	2%	18%	9.12	7%	24%	34.67	5%	9%
18:1n-7c		1.48	2%	18%	1.2	3%	19%	1.35	4%	20%	2.2	5%	19%
20:1n-9c	Gondoic Acid	0.18	7%	23%	0.04	78%	83%	0.2	16%	29%	0.38	8%	24%
24:1n-9c	Nervonic Acid	0.77	17%	32%	0.07	89%	80%	1.39	19%	31%	0.16	97%	233%
	omega-3 polyunsaturated												
18:3n-3c	Alpha-linolenic Acid (ALA)	0.59	4%	36%	0.59	4%	28%	0.23	13%	37%	1.25	7%	40%
20:5n-3c	EPA	0.82	6%	60%	0.91	7%	70%	0.94	10%	59%	0.38	10%	71%
22:5n-3c	DPA	0.5	6%	33%	0.03	52%	57%	0.82	6%	32%	0.42	22%	47%
22:6n-3c	DHA	1.86	7%	34%	0.57	8%	40%	3.05	7%	34%	0.87	13%	53%
	omega-6 polyunsaturated												
18:2n-6cc	Linoleic Acid (LA)	30.35	2%	15%	53.61	1%	10%	21.49	6%	18%	18.4	7%	27%
18:3n-6c	Gamma-linolenic Acid (GLA)	0.49	5%	41%	1.08	16%	42%	0.1	14%	106%	0.46	9%	51%
20:2n-6c		0.27	7%	20%	0.08	98%	80%	0.37	16%	19%	0.36	14%	29%

20:3n-6c	Dihomogammalinolenic Acid (DHGLA)	1.78	4%	23%	0.81	11%	26%	3.11	9%	26%	0.39	22%	32%
20:4n-6c	Arachidonic Acid (AA)	7.51	5%	30%	7.72	5%	34%	10.16	6%	27%	1.62	10%	35%
22:2n-6c		0.04	60%	64%	0.03	136%	107%	0.04	53%	105%	0	NA	136%
22:4n-6c	Adrenic Acid	0.23	6%	26%	0.01	120%	104%	0.37	8%	27%	0.2	10%	33%
trans fatty acids													
14:1n-5t	Myristelaidic Acid	0	NA	138%	0	NA	111%	0.02	69%	88%	0	NA	682%
16:1n-7t	Palmitelaidic Acid	0.18	11%	36%	0.14	31%	39%	0.24	22%	40%	0.14	22%	34%
18:1-trans		0.82	7%	37%	0.29	25%	39%	0.87	16%	33%	1.81	14%	37%
18:2-trans		0.35	10%	21%	0.41	24%	26%	0.3	23%	31%	0.57	13%	23%
18:2n-7c	c9, t11 conjugated linoleic acid (CLA)	0.14	15%	32%	0.11	19%	50%	0.13	38%	41%	0.26	21%	32%
20:1n-9t		0	NA	259%	0	NA	781%	0	NA	334%	0	NA	358%

CE, cholesteryl ester; PL, phospholipid; TG+FFA, triglyceride plus free fatty acids. Plasma lipid fractions separated using silica columns (Sep-Pak® Silica 55-105µm, Glass Syringe 5cc-500mg; Waters Corp Cat# WAT020585).

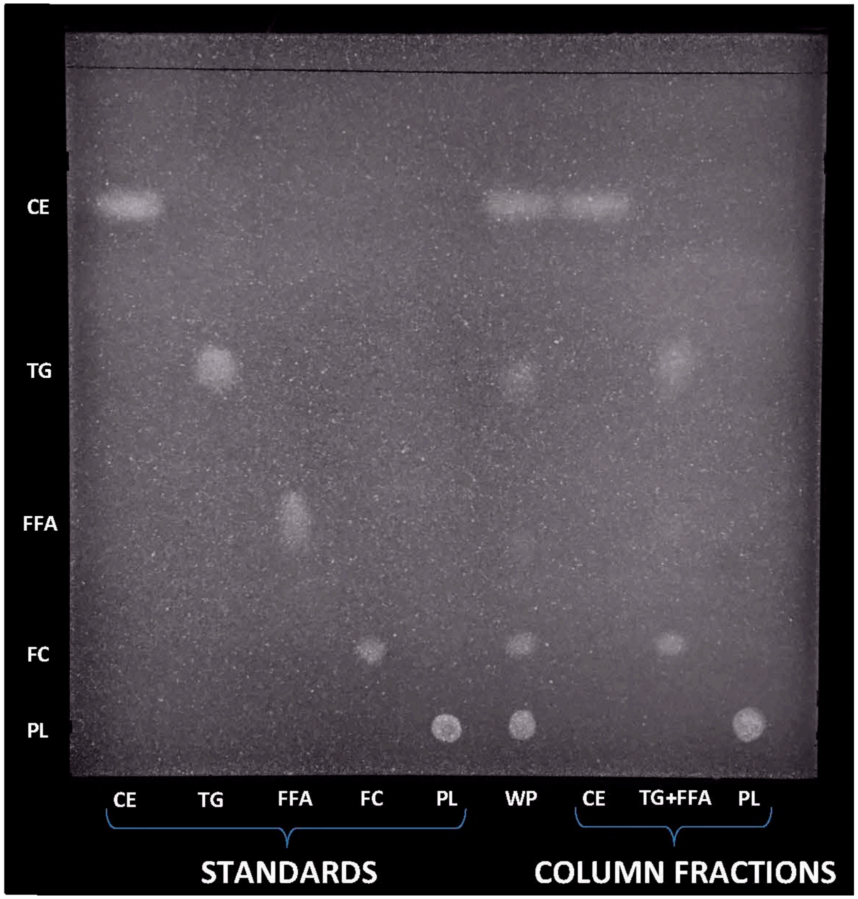

Figure 1. Thin layer chromatography plate (Analtech Uniplate Cat# 02011) confirming successful resolution of plasma fractions by silica columns. From left to right: migration of pure standards of cholesteryl ester (CE; cholesteryl heptadecanoate, NuChek Prep Cat# CH-816), triglyceride (TG; tripentadecanoin, NuChek Prep Cat# T-145), free fatty acid (FFA; pentadecanoic acid, NuChek PrepCat# N-15-A), free cholesterol (FC; NuChek Prep Cat# CH-800), and phospholipid (PL; Phosphotidyl choline; Avanti Polar Lipids Cat# 850360C); whole plasma (WP); and the three plasma fractions prepared by silica columns (Sep-Pak® Silica 55-105μm, Glass Syringe 5cc-500mg; Waters Corp Cat# WAT020585).

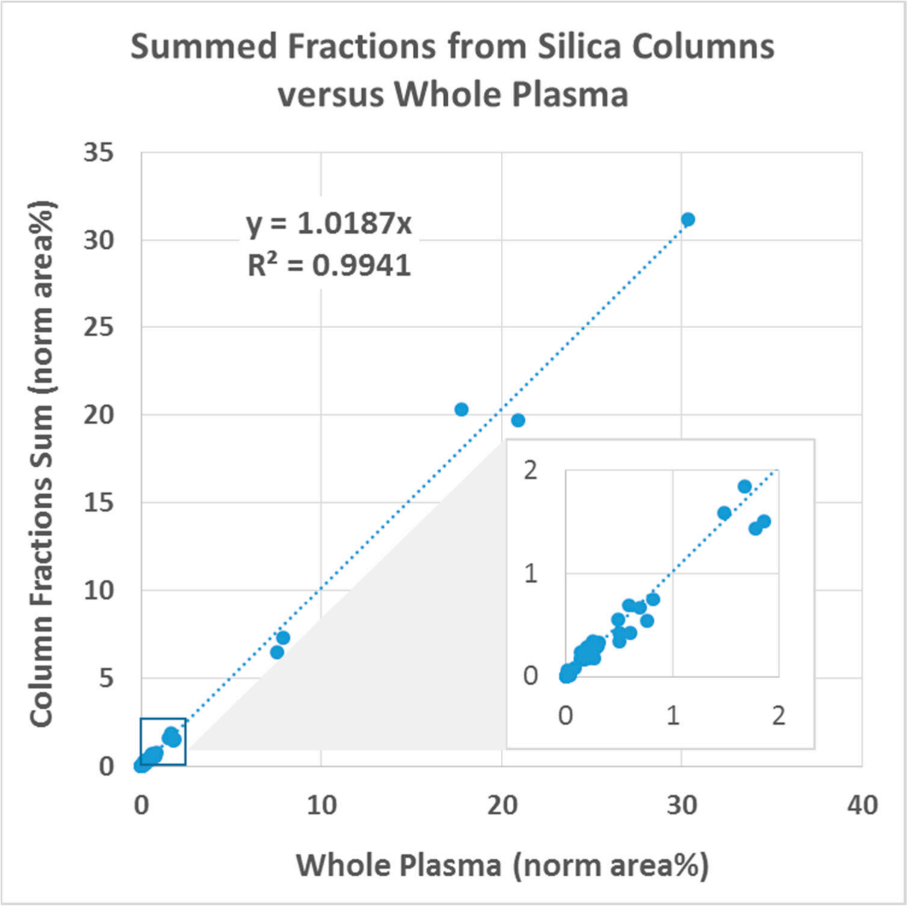

Figure 2. Correlation between the fatty acid levels measured in whole plasma and those obtained from the sum of all fatty acids from the silica column fractions. The smaller graph shows only those fatty acids whose area comprises < 2% of total plasma fatty acids.
